# Interdepartmental Commensality: A Strategy for Increased Interdepartmental Collaboration

**DOI:** 10.5811/westjem.48852

**Published:** 2026-01-24

**Authors:** Jeff Druck, Graham Brant-Zawadzki, Mike Morgan, Jamal Jones, Shilpa Raju, Holden Wagstaff, Emad Awad

**Affiliations:** University of Utah Spencer Fox Eccles School of Medicine, Department of Emergency Medicine, Salt Lake City, Utah

## Abstract

**Introduction:**

The concept of commensality, the act of eating together, is as old as humanity and has been extensively explored in the social sciences and humanities. We sought to assess whether an interdepartmental commensality program would improve cross-departmental familiarity, willingness to engage in scholarly discussions, and enhance collaborative efforts.

**Methods:**

A program was established to arrange dinners for emergency department (ED) faculty with six other departments, after which participants were surveyed about their thoughts on the dinner’s impact. Our primary outcome measure was change in perceived familiarity with interdepartmental colleagues. Secondary outcomes included willingness to engage in academic discussion and perceived likelihood of future collaboration. A program was established to arrange dinners between the ED and six other departments (obstetrics and gynecology, neurology, psychiatry, internal medicine, otolaryngology, and ophthalmology), followed by a post-event survey.

**Results:**

A total of 55 of 81 participants responded to the survey (response rate 67.9%). We found significant increases in familiarity with colleagues (2 pre- to 4/5 post-intervention, P < .001), willingness to discuss academic issues (4 to 5/5, P < .001), and anticipated collaborations (2 to 5/5, P < .001).

**Conclusion:**

An interdepartmental commensality program initiated by an institution’s department of emergency medicine can potentially improve interdepartmental collaboration, familiarity, and discussions.

## INTRODUCTION

The concept of commensality, the act of eating together, is as old as humanity and has been extensively explored in both social science and the humanities.^1^,^2^ However, its potential benefit as a form of relationship-building across disparate medical specialties is relatively uncharted territory. Given the daily interactions of emergency clinicians with multiple specialties, the potential impact of strategies to strengthen interprofessional relationships is significant. This study explores whether something as simple and cost effective as commensality dinners could affect interprofessional relationships and lead to increased collaboration and project-building among colleagues. Although emergency medicine (EM) is not unique in its interactions with multiple specialties, the frequency, timeliness, and nature of these interactions may make it less likely that camaraderie exists within the EM space.

Commensality has been shown to benefit relationship development.^3^,^4^ In this study we explore that concept and examine the potential benefits of commensality dinners between emergency physicians and physicians from other medical specialties. The primary objective was to assess changes in attitudes and perceptions regarding interdepartmental collaboration and communication pre- and post-intervention.

## METHODS

### Study Design

In this study we used a pre-post single-survey experimental design to evaluate the impact of commensality dinners on interdepartmental collaboration and communication with eligible participants, specifically attendees of the scheduled commensality dinners. Participants were selected via a member of each department asking for participants to attend, limited to faculty and fellows; seven members from each department were subsequently scheduled for each dinner. The University of Utah Department of Emergency Medicine (DEM) hosted this series of dinners with various clinical academic departments within our institution, including obstetrics and gynecology, neurology, psychiatry, internal medicine, otolaryngology, and ophthalmology. The food at all dinners was funded by a University of Utah Health Meaningful Use Grant, and the participants’ responsibility was the cost of all beverages. Food choices were limited to a prix fixe menu with accommodations for allergies and sensitivities. There was no preset agenda and no specific topic of conversation that was encouraged for any of the dinners.

We developed a survey instrument using the cognitive response model^5^ and pilot-tested it among EM faculty. It included quantitative and qualitative items to comprehensively understand participants’ attitudes and perceptions. The survey included the following sections:

- Demographics: Primary role in the department (attending/fellow), length of time in the department (1–5 years, 6–10 years, …, 20+ years), and prior involvement in commensality dinners (Yes/No).
- Interdepartmental Collaboration: Assessed using a 5-point Likert scale (1 = Strongly Disagree, 5 = Strongly Agree) to measure perceptions of collaboration, communication, and teamwork between departments.
- Qualitative Feedback: Open-ended questions to gather detailed feedback on participants’ experiences and suggestions for improvement.

Thirty minutes following each dinner, a single survey invitation was sent to participants using standard email, with the responses sent through an anonymous link from Qualtrics (Qualtrics International, Inc, Provo, UT). We performed all data analyses using SPSS v29 (IBM Corporation, Armonk, NY). Descriptive statistics were summarized for baseline characteristics in the entire cohort. We summarized continuous variables as mean, standard deviation, median, and interquartile range based on their distributions. Categorical variables were summarized as frequencies and relative frequencies.

**Population Health Research Capsule**

What do we already know about this issue?

*Interdepartmental collaboration improves care and scholarship, but structured, low-cost strategies to build cross-specialty relationships are limited*.

What was the research question?

*Does an interdepartmental commensality program improve familiarity, academic discussion, and collaboration among faculty?*

What was the major finding of the study?

*Median familiarity increased from 2 to 4 post-intervention (Δ=2, P<.001); expected collaborations rose from 2 to 5 (Δ=3, P<.001)*.

How does this improve population health?

*Stronger interdepartmental relationships may enhance coordinated care, interdisciplinary scholarship, and systems-level improvements across healthcare settings*.

We assessed participants’ familiarity with their colleagues by calculating median scores for pre- and post-intervention periods. The Wilcoxon signed-rank test was used to determine the statistical significance and magnitude of the differences in median scores. Similarly, we computed the median scores for “willingness to discuss academic issues” and “collaborations” before and after the intervention, and the Wilcoxon signed-rank test was used to evaluate potential statistically significant differences in these medians. We also analyzed

secondary outcomes of participant satisfaction, anecdotal reports of actual collaborative activities, gender and departmental roles, and faculty status via the Wilcoxon signed-rank test.

### Participants

Participants included attending physicians and fellows from the DEM and other participating departments. Each departmental contact, who was also a dinner participant, selected the participants, with availability and desire as the two most significant elements that focused on participation.

### Declaration: Ethics, Consent to Participate, and Consent to Publish Declarations

The institutional review board (IRB) at the University of Utah deemed this study as a process improvement project and not specifically research; therefore, it was exempt from IRB review (University of Utah IRB# 00182694). Participation in the survey was voluntary, and informed consent was implied from all participants before data collection via survey completion. We. maintained the participants’ confidentiality and anonymity throughout the study via use of the Qualtrics server database, and the survey was deployed through Qualtrics. This study has not been published in any other form. Data available by request.

## RESULTS

### Study Population

The study included faculty participants from several departments within a central medical school and academic medical center in the United States. Overall participation included 84 faculty members who committed to coming to the dinners; actual attendance was 81, with four people unable to attend due to illness and one additional person attending the dinner unexpectedly. Fifty-five participants responded to the survey, yielding a response rate of 67.9%. As the host department, the DEM was the most represented, with 22 survey respondents, accounting for 40.0% of the respondents. The remainder of the survey respondents were faculty from internal medicine (5 participants, 9.1%), neurology (4 participants, 7.2%), ophthalmology (8 participants, 14.5%), psychiatry (6 participants, 10.9%), and both obstetrics/gynecology and otolaryngology (5 participants each, 9.1%). Regarding the primary roles of the participants, clinical care was the most common role, represented by 26 participants (47.3%). Other roles included education, 15 participants (23.3%); administration, 8 participants (14.5%); and research, 6 participants (10.9%). The mean participant satisfaction score, measured on a scale from 0–100, was notably high at 94.91 ± 8.4. The characteristics of the participants are detailed in Table 1.

### Attendance by Department and Sex

We analyzed attendance at the commensality dinners by department and sex to assess the level of engagement across different fields and to identify any sex-based patterns in participation. Each dinner had participants from the DEM (host department) and one of the guest departments. As previously stated, participants were selected by home departments based on interest and availability. Overall, the sex breakdown in EM was 52% male and 48% female. The breakdown was 74% female and 26% male for all other specialties combined.

### Association of the Commensality Dinners Program and Connection and Collaboration

Participants reported increased familiarity with their colleagues, as evidenced by the Wilcoxon signed-rank test results, which showed a significant median score increase from 2 (disagree) (IQR 1–4) pre-intervention to 4 (agree) (IQR 3–4) post-intervention (median difference = 2, *P* < .001). The willingness to discuss academic issues also saw an improvement from 4 (agree) (IQR 3–4) before the intervention to 5 (strongly agree) (IQR 4–5) afterward (median difference = 1, *P* < .001). Finally, there was a significant association in the number of expected collaborations. The median score for collaborations increased from 2 (disagree) (IQR 1–4) pre-intervention to 5 (strongly agree) (IQR 4–5) post-intervention (median difference = 3, *P* < .001). The association between the commensality dinners program and the study outcomes is summarized in Table 2.

Interestingly, there was no correlation in any variation of response in terms of time as faculty or fellow status, or in terms of role within each department. Similarly, there was no substantial difference in response percentages between the DEM and other departments.

## DISCUSSION

The primary purpose of this study was to evaluate the influence of interdepartmental commensality dinners on fostering collaboration and communication among various clinical academic departments at the University of Utah. The analysis yielded three key outcomes: a marked increase in cross-departmental familiarity; an enhanced willingness to engage in scholarly discussions; and improved collaborative efforts. These findings highlight the potential of informal social events, such as commensality dinners, in cultivating a more cohesive and cooperative work environment in academic medical institutions.

This result highlights the program’s effectiveness in encouraging interdisciplinary collaborations, which is crucial for advancing research and educational outcomes within an academic medical center. Overall, the commensality dinners program positively impacted key metrics of connection, cohesion, and knowledge-sharing among the departments involved. The significant improvements in familiarity, willingness to discuss academic issues, and collaboration underscore the value of such initiatives in fostering a collaborative and cohesive academic community.

As a result of these dinners, a retina camera was purchased, setting up a joint ophthalmology/EM collaborative study. Additionally, a change in staffing for an ED psychiatrist came about, along with cross-collaboration for resident education in a number of the departments. Of note, the provision of funded meals may have influenced participation, as the benefit of a complimentary dinner could have served as a motivating factor for attendance. This study has not yet been replicated under conditions in which participants are responsible for their own meal costs. Our findings align with existing literature highlighting the benefits of informal social interactions in professional settings.^6^–^8^ Previous studies have shown that such interactions can enhance team cohesion, improve communication, and promote collaborative problem-solving.

Despite the small scale of our research, we observed significant statistical improvements across all measured outcomes. These results underscore the importance of investing in social initiatives to bridge interdepartmental divides for policymakers, researchers, and clinicians. By addressing the gap in structured interdepartmental interaction opportunities, this study contributes to the growing body of evidence supporting the role of socialization in professional development and collaboration.

## LIMITATIONS

Despite its positive findings, the study has limitations, including small sample size and potential biases introduced by self-reported data. There was the possibility of reporting bias, in that only respondents who felt the dinner was significant for them may have replied to the survey. Participant attendance may have been influenced by several factors, among them availability during a weeknight dinner, selection by the departmental liaison, and desire to be involved in a group activity. There may have been selection bias, as people more likely to enjoy the event volunteered to be there, or the organizer may have been more persistent with faculty they thought would enjoy the experience more. Another possible source of bias is related to the Hawthorne effect, wherein the fact of being studied varies the participant behavior. Similarly, all the results are from self-reported feelings, which may not correlate into action. Lastly, as this was a retrospective pre-post single survey, there is the possibility of recall bias, with the improvement overemphasized due to the recency of the intervention affecting both the pre- and post-survey results (response-shift bias).

Additionally, the short-term assessment does not necessarily correlate with the long-term sustainability of the program’s benefits, which warrants further investigation. Similarly, the sex findings in this study are striking, although the small sample size and selection method limit the significance of the difference in sex attendance. Possible conclusions may be that the desire for community may be different by specialty or sex, or the makeup of departments may be disparate in their sex makeup. It is difficult to abstract the impact of sex roles from such a small study, but the female sex predominance in non-EM participation deserves further investigation.^9^

The findings of this study are likely to be most applicable to clinical academic staff members, particularly those working in large, multidisciplinary medical centers. The results may not be generalizable to smaller or non-academic institutions where interdepartmental dynamics may differ. Adaptations may be necessary for other settings, especially those with different logistical and financial constraints, to effectively implement similar initiatives. Compared to other interventions to improve interdepartmental collaboration, such as formal team-building workshops or joint academic projects, commensality dinners offer a more relaxed and natural environment for fostering relationships and collaboration.^10^,^11^ The outcomes measured in this study, including familiarity, willingness to discuss academic issues, and collaborations, will likely reflect real-world improvements in interdepartmental relations. These outcomes are measurable in clinical practice and can be replicated in further research to validate the findings.

Based on this study’s findings, future research should focus on assessing the long-term effects of commensality dinners on interdepartmental collaboration and communication. Additionally, investigating the scalability of such programs in different institutional settings could provide insights into their broader applicability.^11^ Examining the impact of similar interventions on patient care outcomes and departmental efficiency could further validate the benefits of fostering social interactions among medical professionals. Finally, developing structured guidelines for implementing commensality programs could help other institutions adopt this strategy to enhance interdepartmental collaboration.

## CONCLUSION

An interdepartmental commensality program between a department of emergency medicine and other departments improved perceived cross-departmental familiarity, willingness to engage in scholarly discussions, and collaborative efforts.

## Figures and Tables

**Table 1:** Descriptive statistics of respondents at interdepartmental commensality dinners.

Variable	Frequency (%)
Sex	
Male	33 (39.3%)
Female	51 (60.7%)
Home department	
Emergency medicine	22 (40.0%)
Internal medicine	5 (9.1%)
Neurology	4 (7.2%)
Obstetrics/gynecology	5 (9.1%)
Ophthalmology	8 (14.5%)
Otolaryngology	5 (9.1%)
Psychiatry	6 (10.9%)
Main Roles	
Administration	8 (14.5%)
Clinical care	26 (47.3%)
Education	15 (23.3%)
Research	6 (10.9%)
Satisfaction (mean ± SD)	94.91 ± 8.4

**Table 2:** Effect of the commensality dinners program on the study outcomes.

Variable	Pre-intervention median (IQR)	Post-intervention median (IQR)	Median difference	P-value
Familiarity with colleagues	2 (1–4)	4 (3–4)	2	< .001
Willingness to discuss academic issues	4 (3–4)	5 (4–5)	1	< .001
Expected collaborations	2 (1–4)	5 (4–5)	3	< .001
